# Healthcare professionals’ perceptions of patient safety culture and teamwork in intrapartum care: a cross-sectional study

**DOI:** 10.1186/s12913-022-08145-5

**Published:** 2022-06-24

**Authors:** Annika Skoogh, Carina Bååth, Marie Louise Hall-Lord

**Affiliations:** 1 Department of Health Sciences, Faculty of Health, Science and Technology, Karlstad University, S-651 88 Karlstad, Sweden; 2 Faculty of Health, Welfare and Organisation, Østfold University College, P.O. Box 700, 1757 Halden, Norway; 3 Department of Health Science, Faculty of Medicine and Health Sciences, Norwegian University of Science and Technology, Teknologivn. 22, 2815 Gjøvik, Norway

**Keywords:** Hospital Survey on Patient Safety Culture, Intrapartum care, Labor ward, Patient safety, Patient safety culture, Perception, Profession, TeamSTEPPS® Teamwork Perceptions Questionnaires, Teamwork

## Abstract

**Background:**

In complex healthcare organizations, such as intrapartum care, both patient safety culture and teamwork are important aspects of patient safety. Patient safety culture is important for the values and norms shared by interprofessional teams in an organization, and such values are principles that guide team members’ behavior. The aim of this study was 1) to investigate differences in perceptions of patient safety culture and teamwork between professions (midwives, physicians, nursing assistants) and between labor wards in intrapartum care and 2) to explore the potential associations between teamwork and overall perceptions of patient safety and frequency of events reported.

**Methods:**

The design was cross-sectional, using the Swedish version of the Hospital Survey on Patient Safety Culture (14 dimensions) and the TeamSTEPPS® Teamwork Perceptions Questionnaire (5 dimensions). Midwives, physicians, and nursing assistants in three labor wards in Sweden in 2018 were included. Descriptive statistics, the Kruskal–Wallis *H* test, two-way ANOVA, and standard multiple regression analysis were used.

**Results:**

The questionnaires were completed by 184 of the 365 healthcare professionals, giving a response rate of 50.4%. Two-way ANOVA showed a significant main effect of profession on two patient safety culture dimensions and one teamwork dimension and a significant main effect of labor ward on four patient safety culture dimensions and four teamwork dimensions. A significant interaction effect of profession and labor ward was found on four patient safety culture dimensions and four teamwork dimensions. The regression analysis revealed that four out of the five teamwork dimensions explained 40% of the variance in the outcome dimension ´Overall perceptions of patient safety´.

**Conclusions:**

The results of the study indicate that profession and labor ward are important for healthcare professionals' perceptions of patient safety culture and teamwork in intrapartum care. Teamwork perceptions are significant for overall patient safety.

## Background

In Sweden, midwives in labor wards play a leading role in anticipated normal childbirths. Midwives usually work in interprofessional teams with nursing assistants and with physicians if complications arise during childbirth [1]. Patient safety culture is viewed as an important organizational aspect that influences patient safety and that is related to teamwork, communication about errors, event reporting and organizational learning [2]. Most definitions of patient safety culture highlight the importance of the values and norms shared by the members of a group, such as a team, profession and organization. Values serve as principles that guide the behaviors of the team members [3]. In the present study, the following definition of patient safety culture was used: ‘An integrated pattern of individual and organizational behavior, based upon shared beliefs and values that continuously seeks to minimize patient harm, which may result from the processes of care delivery’ ([4], p.4). Early work on patient safety culture originates with the report To Err is Human [5] and the framework from the Agency for Healthcare Research and Quality (AHRQ) [6]. A relationship has been demonstrated between a positive patient safety culture and less patient harm [7, 8]. The majority of previous research on patient safety culture has been conducted in hospitals [9–11] and in different types of care contexts, such as surgical care [12], medical care [13] and intensive care [14]. A review by Willmott and Mould [15] found that healthcare professionals had different views of patient safety culture, with physicians perceiving poorer patient safety culture than the other professionals. In intrapartum care, healthcare professionals’ perceptions of patient safety culture have been conducted with midwives [16], other professions with midwives in the minority [17], and with midwives and nurses [18]. To our knowledge, few studies have examined midwives’, physicians’ and nursing assistants’ perceptions about patient safety culture.

The delivery of effective and safe intrapartum care to women is highly dependent on patient-centered collaboration between healthcare professionals working in interprofessional teams [19]. Teamwork is defined by Xyrichis and Ream as ‘a dynamic process involving two or more health professionals with complementary backgrounds and skills, sharing common health goals and exercising concerted physical and mental effort in assessing, planning or evaluating patient care. This is accomplished through independent collaboration, open communication and shared decision-making’ ([20], p.238).

Research on teams and teamwork led to the development of the ‘Big Five’ theoretical framework, which consists of the core components of effective teamwork: team leadership, mutual performance monitoring, backup behavior, adaptability and team orientation [21]. Furthermore, structural issues, such as team composition and task interdependence, and contextual issues, such as leadership and patient safety culture, are important for teamwork performance [22]. The requirement for interprofessional teamwork has increased due to the need for professionals with more complex and specialized knowledge and skills to provide intrapartum care to women. The importance of interprofessional teamwork is also emphasized in guidelines and policy documents [23]. Previous research has shown that teamwork outcomes are often reported at different levels, such the patient, healthcare professional, and healthcare organization levels [20]. Effective teamwork is associated with fewer medical errors [24] and decreased mortality [25]. Lyubovnikova et al. [26] reported that effective teamwork protected patients from harm and created a positive and engaging workplace. Healthcare professionals in labor wards perceived teamwork and open communication to be important for the safe care of women in childbirth [27]. Conversely, a lack of respect, fear of being questioned, experience of not being listened to, and the inability to agree on common safety strategies were identified as barriers to communication and teamwork among healthcare professionals in labor wards [28]. An organizational outcome of teamwork could be the assessment of patient safety culture [29, 30] or workforce and reduced turnover [20]. The majority of previous teamwork research has been conducted in acute care settings, such as operating rooms and trauma units [31].

Patient safety culture and teamwork are key challenges in the delivery and coordination of safe care [22, 32, 33]. A hospital has its own culture at the macro level, and each ward where teams perform their tasks may have its own culture at the micro level [34]. Measurements of healthcare professionals’ perceptions of patient safety culture and teamwork in intrapartum care can be used to raise awareness about patient safety and to identify areas for improvement.

The aim of the study was 1) to investigate differences in perceptions of patient safety culture and teamwork between professions (midwives, physicians, nursing assistants) and between labor wards in intrapartum care and 2) to explore the potential associations between teamwork and overall perceptions of patient safety and frequency of events reported.

## Methods

### Design

The study had a cross-sectional design.

### Setting and sample

The study was conducted in four labor wards in four hospitals in three regions in Sweden. Two labor wards in one region had the same head manager and were merged into one labor ward. Table 1 displays the number of births and number of healthcare professionals in the labor wards (Labor ward 1, Labor ward 2, Labor ward 3). Healthcare professionals (*N* = 365), including midwives (*n* = 186), physicians (*n* = 107) and nursing assistants (*n* = 72) working in labor wards, were invited to participate in the study. The inclusion criterion was frontline healthcare professionals. The exclusion criteria were healthcare professionals on sick leave or parental leave.

**Table 1:** Number of births and number of healthcare professionals in the labor wards in 2018

	**Labor ward 1**	**Labor ward 2**	**Labor ward 3**
**Number of births**	2879	3600	3430
**Number of healthcare professionals**			
Midwives	47	75	64
Physicians	37	37	33
Nursing assistants	21	27	24

### Measurements

The measurements included two questionnaires and four background questions.

The Swedish version of the Hospital Survey on Patient Safety Culture (S-HSOPS) [35] was used to measure healthcare professionals’ perceptions of patient safety culture. The HSOPS was designed by the AHRQ; it consists of 42 items in 12 dimensions (seven unit-level dimensions, three hospital-level dimensions, two outcome dimensions) and two outcome items [36]. The Swedish version of the questionnaire consists of two additional dimensions with six items and one additional outcome item [35] (Table 2).

**Table 2:** The S-Hospital Survey on Patient Safety Cultures dimensions, outcome items and number of items

Dimensions	Items
**Unit level dimensions**	
Communication openness	3
Feedback and communication about error	3
Nonpunitive response to error	3
Organizational learning – continuous improvement	3
Staffing	4
Supervisor/manager expectations and actions promoting patient safety	4
Teamwork within units	4
Information and support to patients and family who have suffered an adverse event^1^	4
Information and support to staff who have been involved in an adverse event^1^	2
**Hospital level dimensions**	
Handoffs and transitions	4
Management support for patient safety	3
Teamwork across units	4
**Outcome dimensions**	
Frequency of events reported	3
Overall perceptions of patient safety	4
Outcome items	
Patient safety grade	1
Number of events reported	1
Number of risks reported^1^	1

^1^Additional dimensions and items in the Swedish version

The items in the dimensions are answered on a five-point Likert scale from 1 = ‘Strongly disagree’ to 5 = ‘Strongly agree’, or from 1 = ‘Never’ to 5 = ‘Always’. The outcome item ‘Patient safety grade’ is rated from 1 = ‘Failing’ to 5 = ‘Excellent’. The outcome item ‘Number of events reported’ is rated as follows: 1 = ‘No event’, 2 = ‘1–2 events’, 3 = ‘3–5 events’, 4 = ‘6–10 events’, 5 = ‘11–20 events’, and 6 = ‘ ≥ 21 events’. The additional outcome item ‘Number of risks reported’ in the S-HSOPS is rated 1 = ‘No risk’, 2 = ‘1–2 risks’, 3 = ‘3–5 risks’, 4 = ‘6–10 risks’, 5 = ‘11–20 risks’ and 6 = ‘ > 21 risks’. Eighteen negatively worded items were reversed. The mean score of the items in each dimension was computed to a total score and divided by the number of items in the dimension [36]. The HSOPS and S-HSOPS have been tested for psychometric properties [35, 37].

The TeamSTEPPS® Teamwork Perceptions Questionnaire (T-TPQ) was used to measure healthcare professionals’ individual perceptions of teamwork at the group level. The T-TPQ is a part of the Team Strategies and Tools to Enhance Performance and Patient Safety® (TeamSTEPPS®) package, which is based on the ‘Big Five’. The T-TPQ consists of five dimensions: ‘Team structure’ and the teamwork competencies ‘Leadership’, ‘Situation monitoring’, ‘Mutual support’ and ‘Communication’ [38]. Each dimension includes seven items measured on a five-point Likert scale from 1 = ‘Strongly disagree with the statement’ to 5 = ‘Strongly agree with the statement’. The mean scores of the items in each dimension were computed to a total score and divided by the number of items in the dimension [38]. The T-TPQ has been tested for reliability and validity [38, 39] and translated to Swedish and tested for reliability and construct validity [40].

The four background questions were on profession, age, work experience in the ward, and hours worked per week.

### Data collection

The data collection took place between September and December 2018. The first author informed healthcare professionals in the labor wards about the study. The chief managers administered the questionnaires, including information letters, in paper form to the healthcare professionals. They also reminded the healthcare professionals to participate in the study by e-mail and during meetings. Completed questionnaires were returned anonymously in preprinted envelopes.

### Data analysis

IBM SPSS Statistics, version 25, was used to analyze the data. Descriptive statistics displaying frequency, percentage, mean and standard deviation were calculated. A Kruskal–Wallis *H* test was used to analyze differences between professions and between labor wards according to background questions, with a *p* level of < 0.05 considered statistically significant. The Kruskal–Wallis *H* test was also used to analyze differences between outcome items in the S-HSOPS.

A general linear model analysis with a two-way ANOVA was conducted to explore the main and interaction effects of the profession and labor ward variables on the T-TPQ and S-HSOPS dimensions [41, 42]. A *p* value of < 0.05 was set for the analysis. Levene’s test examined whether the variability in scores for each of the groups was similar (*p* > 0.05). When Levene’s test was significant, a more stringent significance level was set (*p* < 0.01) [41]. Tukey’s HSD post-hoc test was carried out to identify differences between groups when ANOVA was significant. The effect size with partial eta squared was calculated, applying the criterion by Cohen: 0.01 = small effect, 0.06 = moderate effect, and 0.14 = large effect [43].

A standard multiple regression analysis [42] was run to explore potential associations between teamwork dimensions (‘Team structur’, ‘Leadership’, ‘Situation monitoring’, ‘Mutual support’, ‘Communication’) as independent variables and the outcome dimensions of patient safety culture (‘Overall perceptions of patient safety’, ‘Frequency of events reported’) as dependent variables. A *p* level of < 0.05 was considered statistically significant.

## Results

The questionnaire was completed by 184 of the 365 healthcare professionals, giving a response rate of 50.4%. In total, 103 out of 186 (55.4%) midwives, 44 out of 107 (44.1%) physicians and 30 out of 72 (41.7%) nursing assistants participated. Seven healthcare professionals did not respond to the question on profession, which means that they were excluded from the analyses that related to the question on profession.

Comparisons of background characteristics between the professions and between the labor wards are presented in Table 3. Statistically significant differences were found between the professions. The physicians had the highest number of hours worked per week. Comparisons between the labor wards showed a significant difference in the ages of healthcare professionals. The healthcare professionals in Labor ward 2 were the youngest (Table 3).

**Table 3:** Healthcare professionals’ background characteristics in relation to profession and labor ward (*n* = 184)

	**Midwives (*n* = 103)**	**Physicians (*n* = 44)**	**Nursing assistants (*n* = 30)**	**Missing**	**Kruskal–Wallis*H***	***P* value**
	**Mean (SD)**	**Mean (SD**)	**Mean (SD)**			
Age	47.0 (11.10)	43.0 (11.03)	48.1 (12.19)	7	5.56	.062
Work experience in the ward	12.9 (10.45)	8.3 (7.71)	11.6 (10.22)	8	5.55	.063
Hours worked per week	35.8 (4.15)	43.2 (4.13)	34.7 (6.50)	13	69.46	.000
	**Labor ward 1 (*n* = 57)**	**Labor ward 2 (*n* = 72)**	**Labor ward 3 (*n* = 55)**	**Missing**	**Kruskal–Wallis*H***	***P*value**
	**Mean (SD)**	**Mean (SD)**	**Mean (SD)**			
Age	47.9 (12.42)	43.4 (10.19)	49.1 (11.76)	7	9.23	.010
Work experience in the ward	14.6 (12.35)	9.6 (8.33)	11.3 (8.99)	8	3.58	.167
Hours worked per week	38 (6.19)	37.1 (5.95)	37.1 (6.02)	13	1.51	.471

Six of the healthcare professionals were excluded from further analysis of the HSOPS questionnaire (2 midwives, 2 nursing assistants and 2 who did not respond to the question on profession), because of an internal dropout rate of more than 50% of the items. Two of the healthcare professionals were excluded from further analysis of the T-TPQ (1 midwife and 1 who did not respond to the question on profession) because of an internal dropout rate of more than 50% of the items.

A two-way ANOVA showed a significant main effect of profession on two patient safety culture dimensions (‘Staffing’, ‘Information and support to patients and family who have suffered an adverse event’) and one teamwork dimension (‘Team structure’). The effect size was moderate or close to moderate (Table 4).

**Table 4:** Main effect by two-way ANOVA between profession

	Midwives (M) *n* = 101	Physicians (P) *n* = 44	Nursing assistants (NA) *n* = 28		*F*	*P* value	Tukey’s HSD	Effect size^5^
	Mean (SD)	Mean (SD)	Mean (SD)					
**S-Hospital Survey on Patient Safety Culture**								
**Unit level dimensions**								
Communication openness^1^	3.7 (.63)	3.7 (.60)	3.7 (.64)		*F(*2,164) = .10	.903		
Feedback and communication about error^1^	3.9 (.67)	3.6 (.70)	3.9 (.81)		*F*(2,164) = 1.37	.258		
Nonpunitive response to error^2^	3.8 (.77)	3.8 (.81)	3.8 (.71)		*F*(2,164) = .33	.722		
Organizational learning – continuous improvement^2^	3.6 (.58)	3.7 (.62)	3.8 (.74)		*F*(2,164) = .87	.423		
Staffing^2^	3.3 (.89)	2.9 (.99)	3.6 (.65)		*F*(2,165) = 5.16	.007^4^	NA > P (*p* = .002)	.06
Supervisor/manager expectations and actions promoting patient safety^2^	3.7 (.84)	3.7 (.67)	4.1 (.78)		*F*(2,164) = 1.46	.235		
Teamwork within units^2^	4.3 (.51)	4.1 (.46)	4.3 (.59)		*F*(2,165) = 1.96	.144		
Information and support to patients and family who have suffered an adverse event^2^	3.8 (.58)	4.1 (.64)	3.9 (.73)		*F*(2.165) = 4.73	.010	P > M (*p* = .012)	.05
Information and support to staff who have been involved in an adverse event^2^	3.5 (.87)	3.6 (.85)	4.0 (.95)		*F*(2,164) = 2.15	.120		
**Hospital level dimensions**								
Handoffs and transitions^2^	3.7 (.63)	3.5 (.80)	3.8 (.73)		*F*(2,166) = 1.20	.303		
Management support for patient safety^2^	2.8 (.85)	3.2 (.83)	3.0 (.80)		*F*(2,165) = 2.70	.070		
Teamwork across units^2^	3.4 (.68)	3.7 (.60)	3.5 (.86)		*F*(2,166) = 2.88	.059		
**Outcome dimensions**								
Frequency of events reported^1^	3.2 (.76)	3.1 (.87)	3.5 (.87)		*F*(2,163) = 2.32	.101		
Overall perceptions of patient safety^2^	3.8 (.67)	3.7 (.78)	4.1 (.69)		*F*(2,165) = 2.88	.059		
**TeamSTEPPS® Teamwork Perceptions Questionnaire**	*n* = 102	*n* = 44	*n* = 30					
Team Structure^3^	4.0 (.61)	3.7 (.83)	4.3 (.67)		*F*(2,168) = 7.21	.001	NA > P (*p* = .000) M > P (*p* = .022)	.08
Leadership^3^	3.5 (.93)	3.8 (.73)	4.1 (.97)		*F*(2,168) = 3.03	.051		
Situation monitoring^3^	4.0 (.58)	3.7 (.59)	3.9 (.72)		*F*(2,167) = 1.62	.202		
Mutual support^3^	3.9 (.62)	3.6 (.69)	3.9 (.71)		*F*(2,167) = 2.27	.107		
Communication^3^	4.0 (.57)	3.7 (.68)	3.9 (.62)		*F*(2,168) = 2.05	.132		

^1^Scale ranged from 1 = ‘Never’ to 5 = ‘Always’

^2^Scale ranged from 1 = ‘Strongly disagree’ to 5 = ‘Strongly agree’

^3^Scale ranged from 1 = ‘Strongly disagree with the statement’ to 5 = ‘Strongly agree with the statement’

^4^Levene’s test was significant: *p* < 01

^5^Effect size with partial eta squared

The two-way ANOVA demonstrated a significant main effect of labor wards on four patient safety culture dimensions (‘Feedback and communication about error’, ‘Nonpunitive response to error’, ‘Organizational learning – continuous improvement’, ‘Teamwork across units’) and on four teamwork dimensions (‘Team structure’, ‘Leadership’, ‘Situation monitoring’, ‘Communication’). The effect size was moderate in just over half of the cases and otherwise small (Table 5).

**Table 5:** Main effect by two-way ANOVA between labor ward

	Labor ward 1 *n* = 54	Labor ward 2 *n* = 72	Labor ward 3 *n* = 52	*F*	*P* value	Tukey’s HSD	Effect size^5^
	Mean (SD)	Mean (SD)	Mean (SD)				
**S-Hospital Survey on Patient Safety Culture**							
**Unit level dimensions**							
Communication openness^1^	3.6 (.60)	3.7 (.65)	3.9 (.61)	*F*(2,164) = 2.39	.095		
Feedback and communication about error^1^	3.5 (.67)	4.0 (.70)	3.9 (.67)	*F*(2,164) = 9.07	.000	2 > 1 (*p* = .001) 3 > 1 (*p* = .008)	.10
Nonpunitive response to error^2^	3.6 (.74)	3.9 (.76)	3.7 (.80)	*F*(2,164) = 4.01	.020	2 > 1 (*p* = .014)	.05
Organizational learning – continuous improvement^2^	3.4 (.55)	3.7 (.64)	3.8 (.60)	*F*(2,164) = 8.97	.000	2 > 1 (*p* = .001) 3 > 1 (*p* = .001)	.10
Staffing^2^	3.1 (.78)	3.3 (1.05)	3.3 (.81)	*F*(2,165) = 1.13	.326		
Supervisor/manager expectations and actions promoting patient safety^2^	3.7 (.68)	3.6 (.93)	4.0 (.67)	*F*(2,164) = 2.28	.106		
Teamwork within units^2^	4.2 (.48)	4.3 (.63)	4.3 (.42)	*F*(2,165) = 3.57	.031	Not significant	
Information and support to patients and family who have suffered an adverse event^2^	3.9 (.64)	4.0 (.63)	3.9 (.63)	*F*(2,165) = 4.45	.013	Not significant	
Information and support to staff who have been involved in an adverse event^2^	3.5 (.93)	3.7 (.87)	3.8 (.87)	*F*(2,164) = 2.18	.116		
** Hospital level dimensions**							
Handoffs and transitions^2^	3.4 (.71)	3.9 (.58)	3.6 (.73)	*F*(2,166) = 4.62	.011^4^		
Management support for patient safety^2^	3.2 (.72)	2.8 (.87)	2.9 (.87)	*F*(2,165) = 2.71	.069		
Teamwork across units^2^	3.5 (.58)	3.3 (.75)	3.7 (.66)	*F*(2,166) = 5.22	.006^4^	3 > 2 (*p* = .002)	.06
**Outcome dimensions**							
Frequency of events reported^1^	3.1 (.74)	3.3 (.88)	3.3 (.82)	*F*(2,163) = 1.71	.185		
Overall perceptions of patient safety^2^	3.7 (.71)	3.8 (.79)	3.8 (.58)	*F*(2,165) = .59	.555		
**TeamSTEPPS® Teamwork Perceptions Questionnaire**	*n* = 57	*n* = 71	*n* = 55				
Team Structure^3^	3.8 (.69)	4.1 (.75)	3.9 (.59)	*F*(2,168) = 3.07	.049	2 > 1 (*p* = .011)	.04
Leadership^3^	3.4 (.76)	3.5 (1.03)	4.1 (.74)	*F*(2,168) = 8.67	.000^4^	3 > 1 (*p* = .000) 3 > 2 (*p* = .001)	.09
Situation monitoring^3^	3.9 (.59)	4.1 (.66)	3.7 (.54)	*F*(2,167) = 4.87	.009	2 > 3 (*p* = .003)	.06
Mutual support^3^	3.7 (.64)	3.9 (.76)	3.8 (.53)	*F*(2,167) = 2.68	.072		
Communication^3^	3.8 (.65)	4.1 (.59)	3.8 (.56)	*F*(2,168) = 4.68	.011	2 > 1 (*p* = .006) 2 > 3 (*p* = .007)	.05

^1^Scale ranged from 1 = ‘Never’ to 5 = ‘Always’

^2^Scale ranged from 1 = ‘Strongly disagree’ to 5 = ‘Strongly agree’

^3^Scale ranged from 1 = ‘Strongly disagree with the statement’ to 5 = ‘Strongly agree with the statement’

^4^Levene’s test was significant: *p* < .01

^5^Effect size with partial eta squared

A significant interaction effect was found on four patient safety culture dimensions, namely, ‘Staffing’, ‘Supervisor/manager expectations and actions promoting patient safety’, ‘Information and support to staff who have been involved in an adverse event´ and ´Overall perceptions of patient safety’ (S-HSOPS), and four teamwork dimensions, namely, ‘Team structure’, ‘Situation monitoring’, ‘Mutual support’ and ‘Communication’ (T-TPQ). The effect size was moderate (Table 6).

**Table 6:** Interaction effect by two-way ANOVA between profession and labor ward

	*F*	*P* value	Effect size^1^
**S-Hospital Survey on Patient Safety Culture**			
**Unit level dimensions**			
Staffing	*F*(4,165) = 4.37	.002	.10
Supervisor/manager expectations and actions promoting patient safety	*F*(4,164) = 3.04	.019	.07
Information and support to staff who have been involved in an adverse event	*F*(4,164) = 2.82	.027	.06
**Outcome dimensions**			
Overall perceptions of patient safety	*F*(4,165) = 2.94	.022	.07
**TeamSTEPPS® Teamwork Perceptions Questionnaire**			
Team structure	*F*(4,168) = 2.62	.037	.06
Situation Monitoring	*F*(4,167) = 2.76	.030	.06
Mutual Support	*F*(4,167) = 3.58	.008	.08
Communication	*F*(4,168) = 2.86	.025	.06

^1^Effect size with partial eta squared

A Kruskal–Wallis *H* test showed that the outcome items ‘Number of events reported’ and ‘Number of risks reported’ were significantly different, which is shown in Table 7. Physicians scored the highest in ‘Number of events reported’, and the healthcare professionals in Labor ward 2 scored the lowest in ‘Number of risks reported’.

**Table 7:** Differences in outcome items (S-HSOPS) between profession and between labor ward

	**Midwives (*n* = 101)**	**Physicians (*n* = 44)**	**Nursing assistants (*n* = 28)**	**Kruskal–Wallis*H***	***P*value**
	**Mean (SD)**	**Mean (SD)**	**Mean (SD)**		
Patient safety grade^1^	2.1 (.66)	2.3 (.71)	2.1 (.90)	3.32	.190
Number of events reported^2^	1.9 (.68)	2.2 (.90)	1.3 (.72)	21.34	.000
Number of risks reported^3^	1.6 (.91)	1.6 (.92)	1.3 (.71)	4.554	.103
	**Labor ward 1 (*n* = 54)**	**Labor ward 2 (*n* = 70)**	**Labor ward 3 (*n* = 52)**	**Kruskal Wallis*H***	***P*value**
	**Mean (SD)**	**Mean (SD)**	**Mean (SD)**		
Patient safety grade^1^	2.3 (.77)	2.1 (.77)	2.2 (.54)	2.29	.319
Number of events reported^2^	1.9 (.83)	1.7 (.70)	1.8 (.86)	1.10	.576
Number of risks reported^3^	1.7 (.92)	1.4 (.76)	1.7 (.96)	7.73	.021

^1^Scale ranged from 1 = ‘Failing’ to 5 = ‘Excellent’

^2^Scale ranged from 1 = ‘no event’, 2 = ‘1–2 events’, 3 = ‘3–5 events’, 4 = ‘6–10 events’, 5 = ‘11–20 events’, 6 = ‘ ≥ 21 events’

^3^Scale ranged from 1 = ‘no risk’, 2 = ‘1–2 risks’, 3 = ‘3–5 risks’, 4 = ‘6–10 risks’, 5 = ‘11–20 risks’ and 6 = ‘ > 21 risks’

A standard multiple regression analysis revealed that four out of the five teamwork dimensions explained 40% of the variance of the outcome dimension ‘Overall perceptions of patient safety’ (‘Team structure’ B = 0.287, *p* = 0.000; ‘Leadership’ B = 0.253, *p* = 0.000; ‘Mutual support’ B = 0.181, *p* = 0.043; ‘Communication’ B = 0.173, *p* = 0.021). One of the teamwork dimensions explained 8% of the variance of the outcome dimension ‘Frequency of events reported´ but did not reach statistical significance (‘Mutual support’ B = 0.206, *p* = 0.061).

## Discussion

The results showed overall positive perceptions of patient safety culture and teamwork. The two-way ANOVA showed significant differences between professions and between labor wards in relation to patient safety culture and teamwork. Four dimensions of the S-HSOPS and four dimensions of the T-TPQ showed an interaction effect with moderate effect size.

The dimension ‘Teamwork within units’ had the highest score of the dimensions of the S-HSOPS, which is in line with other studies [44, 45]. This dimension was found to be one of the predictors for ‘Overall perceptions of patient safety’ in intensive care [14]. High scores on this dimension indicate a ward where healthcare professionals support each other, treat each other with respect and work together as a team [36].

‘Staffing’ was one of the dimensions in the S-HSOPS with a low score, which is consistent with other studies [11, 44–46]. The physicians had a significantly lower score than the nursing assistants in this dimension. The physicians had a high workload, with the highest number of work hours per week, and may therefore perceive that there were not sufficient healthcare professionals to provide the best care for women undergoing childbirth.

The ‘Staffing’ dimension scored low for all professions. This dimension affects the management of unwanted variation in the complexity of women giving birth and related unexpected situations. To effectively manage unwanted variation [47] in the complex work system [48] of intrapartum care can be solved through staff density. Furthermore, activities that aim to increase risk awareness and preparedness, such as taking into account long-term consequences for patient safety in planning and prioritization decisions and having good foresight regarding the needed supply of healthcare professionals skills, should be a leadership consideration [49].

Of the dimensions in the T-TPQ, only ‘Team structure’ was significantly different between the professions, with the physicians scoring the lowest. ‘Team structure’ refers to organizational structures, healthcare professionals’ roles and responsibilities and the ward’s goals, efficiency, and resources to ensure patient safety [38]. The lower perceived score from the physicians in this study may indicate unfulfilled expectations in team structures regarding system components to ensure patient safety. Research shows that structural issues, such as team composition, are important for teamwork performance and serve as the basis for improvement [22].

The healthcare professionals in Labor ward 2 scored significantly higher than the healthcare professionals in Labor ward 1 on three S-HSOPS dimensions, including ‘Feedback and communication about error’ and ‘Organizational learning – continuous improvement’. ‘Feedback and communication about error’ refers to being informed about errors and adverse events that happen, providing feedback about changes implemented and enablers’ opportunities to discuss how to prevent errors and adverse events [36]. The healthcare professionals in Labor ward 1 seemed to be least satisfied with how adverse events were handled in the organization.

There was no significant difference between either the professions or labor wards concerning the ‘Frequency of events reported’ dimension, while physicians and midwives scored higher than did nursing assistants on the item ‘Number of events reported’. This result is probably due to midwives and physicians filling out and submitting event reports to a greater degree than nursing assistants, since it is more common that these two professions perform treatments in the labor ward that can lead to subsequent events compared to nursing assistants. The healthcare professionals in Labor ward 2 responded significantly lower than did the healthcare professionals in other labor wards on the outcome item ‘Number of risks reported’, even though they had positive scores for ‘Feedback and communication about error’ and ‘Organizational learning – continuous improvement’. This outcome can be seen as contradictory and difficult to explain. The Swedish Patient Safety Act [50] states that healthcare professionals should report risks that an event could possibly occur.

An incident reporting culture is a part of the patient safety culture and creates opportunities for organizational learning and improved patient safety [36]. Voluntary patient safety incident reporting has commonly been found to be a foundation for patient safety work. However, voluntary patient safety incident reporting suffers from underreporting and lack of organizational learning [51]. The dimension ‘Organizational learning – continuous improvement´ refers to mistakes that have led to positive changes and changes that have subsequently been evaluated for effectiveness [36]. This dimension has been found to be a predictor of the incident reporting culture in hospital units [52]. An interview study found that physicians had expectations of being infallible, which reduced their willingness to speak about errors they made, thus limiting opportunities for learning from adverse events [53].

Perceptions of teamwork (T-TPQ) differed significantly between the labor wards in four out of five dimensions. The healthcare professionals in Labor ward 3 had the highest score on the ‘Leadership’ dimension. This dimension concerns the ability to maximize the activities of team members by ensuring that team actions are understood and that changes in information are shared, given that the team members have the necessary resources [38]. In intrapartum care, leadership is both important and challenging. Most childbirths do not incur complications and adverse events [19], but any childbirth can rapidly deteriorate and require prompt interventions by the team [54].

The ‘Situation monitoring’ and ‘Communication’ dimensions had the highest scores in Labor ward 2. The team competence ‘Situation monitoring’ reflects the process of actively scanning and assessing situational elements to gain information or understanding or to maintain awareness to support team functioning [36]. A tolerant atmosphere in the labor ward leads team members to exchange relevant information and dare to speak up to strengthen patient safety [27]. ‘Communication’ is the structured process by which information is clearly and accurately exchanged among team members [38]. In labor wards, using the SBAR (Situation, Background, Assessment, Recommendation) at handovers and during rounds decreased the risk for missing information [27]. The differences between the labor wards are difficult to interpret. The Swedish Medical Birth Register indicates that interventions in intrapartum care differ among regions in Sweden. An example is the prevalence of cesarean section, which varies between 8 and 25% [55]. A cesarean section increases the risk for adverse events for both the woman and the child [56]. The Swedish patient safety plan highlights that a positive patient safety culture is an important condition for safe care in the healthcare organization [49].

The regression analysis revealed that four of the teamwork dimensions (‘Team structure’, ‘Leadership’, ‘Mutual support’, ‘Communication’) were associated with the outcome dimension ‘Overall perceptions of patient safety’. This result indicates how important these teamwork competencies are for procedures and systems to prevent errors and patient safety problems. Team training can be used to increase healthcare professionals’ perceptions of patient safety culture and to improve teamwork in intrapartum care [29]. Team and team performance is central in complex healthcare and in system theory about patient safety [48]. Team training can improve the effectiveness of interprofessional teams [31, 57] in terms of teamwork performance [58] and has positive effects on patient safety culture [57], clinical processes and a reduction in adverse events [59].

## Limitations

A limitation was the relatively low response rate. Nevertheless, a response rate of at least 50% is preferable [36], which was achieved in this study. Unfortunately, no dropout analysis could be carried out for those who did not reply because the questionnaire was answered anonymously. A possible source of selection bias could be that more interested and thereby more positive healthcare professionals participated in the study. Another limitation was that Levene´s test had significant indications in the first step of the two-way ANOVA in a few analyses, which suggests that the variance in the dependent variable across the groups was not equal [41]. However, the standard deviations in the dependent variables were not spread out in relation to the mean scores.

## Conclusion

The results of this study indicate that profession and labor ward are important for healthcare professionals' perceptions of patient safety culture and teamwork in intrapartum care. The differences between the labor wards seemed to have a greater impact than the differences between the professions. Teamwork perceptions were shown to be significant for overall patient safety. Future studies are necessary to enhance the knowledge from this study to a wider population of frontline healthcare professionals concerning patient safety culture and teamwork in labor wards.

## Data Availability

The datasets generated and/or analyzed during the current study are not publicly available due to privacy and confidentiality agreements but are available from the corresponding author on reasonable request.

## Abbreviations

AHRQ: Agency for Healthcare Research and Quality
ANOVA: Analysis of variance
HSOPS: Hospital Survey on Patient Safety Culture
S-HSOPS: Swedish version of the Hospital Survey on Patient Safety Culture
SBAR: Situation, Background, Assessment, Recommendation
TeamSTEPPS®: Team Strategies and Tools to Enhance Performance and Patient Safety
T-TPQ: TeamSTEPPS® Teamwork Perceptions Questionnaire
Tukey’s HSD: Tukey’s honest significant difference
